# Reviving Movement and Stability: A Case Series on Different Innovative Rehabilitation Strategies Post-anterior Cruciate Ligament Reconstruction

**DOI:** 10.7759/cureus.67730

**Published:** 2024-08-25

**Authors:** Nikita Gangwani, Gurjeet Kaur, Pratik Phansopkar

**Affiliations:** 1 Musculoskeletal Physiotherapy, Ravi Nair Physiotherapy College, Datta Meghe Institute of Higher Education & Research (Deemed to be University), Wardha, IND; 2 Centre for Advance Physiotherapy Education and Research, Ravi Nair Physiotherapy College, Datta Meghe Institute of Higher Education & Research (Deemed to be University), Wardha, IND

**Keywords:** case series, physiotherapy, ligamentous knee injury, musculoskeletal rehabilitation, arthroscopic acl reconstruction

## Abstract

Injuries to the anterior cruciate ligament (ACL) are frequent and can seriously impair stability and mobility. This study examines rehabilitation outcomes in four patients following ligament reconstruction. Four patients who underwent ACL reconstruction and received different physiotherapy protocols, namely, "Oxford Knee Services," "Mass General Brigham," "Fowler Kennedy Sports Medicine," and "Schlechter Protocol of Youth Sports and Ortho," were included. The study aimed to identify the most effective rehabilitation approach. Demographic data, injury details, clinical examinations, and preoperative investigations were presented. Outcome measures included pain scores, range of motion (ROM), muscle strength, and functional assessments. All the patients showed improvements, but the rate of progress varied. Patient 3 achieved the best results in the ROM, muscle strength, and functional measures. This suggests that individual factors and rehabilitation protocols might influence outcomes. This study highlights the varying impacts of different rehabilitation protocols on the recovery outcomes of the patients’ post-ACL reconstruction. Despite all patients showing improvements in pain reduction, ROM, muscle strength, and functional capabilities, the rate of progress and the degree of improvement differed notably among them.

## Introduction

Anterior cruciate ligament (ACL) injuries are among the most common knee ligament injuries, with approximately 200,000 cases reported annually worldwide [1]. This high incidence highlights the significance of ACL injuries in both athletic and general populations [2]. The ACL plays a crucial role in knee stability by limiting the forward motion of the tibia and restricting excessive rotational movements [3]. An ACL injury compromises joint integrity and increases the risk of meniscal and cartilage damage, which may lead to osteoarthritis later in life [4]. ACL injuries typically occur during activities that involve sudden changes in direction, abrupt stops, or awkward landings, making the ligament particularly vulnerable in sports [5]. Patients often experience a distinctive "pop" sound at the time of injury, followed by intense pain, rapid swelling, and a loss of range of motion [6]. Instability, especially during pivoting or twisting movements, is also a common symptom [7].

Treatment approaches for ACL injuries have evolved significantly, with both surgical and non-surgical options available [8]. While non-operative management can be effective, it often falls short in younger, active patients due to persistent instability and the risk of further joint damage [9]. ACL reconstruction (ACLR) and repair are surgical options, with ACLR being more common. ACLR involves replacing the damaged ligament with grafts, whereas repair focuses on suturing the torn ACL ends.

Regardless of the chosen treatment, physical therapy is critical for recovery, aiding in the restoration of strength, range of motion, and overall function. Various rehabilitation protocols exist, each differing in their approach to timing, duration, and specific interventions. In this case series, we analyzed four patients who underwent ACL reconstruction, each following a distinct rehabilitation protocol: the "Oxford Knee Services" [10], "Mass General Brigham" [11], "Fowler Kennedy Sports Medicine" [12], and "Schlechter Protocol of Youth Sports and Ortho" [13]. Our goal was to assess the effectiveness of these protocols in postoperative recovery, providing insights into the most beneficial approach for optimal outcomes.

## Case presentation

This case series includes four consecutive patients diagnosed with ACL tears, who were referred to Acharya Vinoba Bhave Rural Hospital, Wardha, India. Following their ACLR surgeries, they each began physiotherapy sessions starting from the postoperative day, but with different rehabilitation protocols. The patients’ data included demographic information and medical history, which are presented in Table 1.

**Table 1:** Demographic details of the patients ACL: anterior cruciate ligament

	Patient 1	Patient 2	Patient 3	Patient 4
Age	22 years	23 years	24 years	24 years
Sex	Male	Male	Male	Male
Occupation	Student	Student	Student	Student
Side affected	Left	Right	Left	Left
Type of injury	Traumatic and grade 3 type of ACL tear	Traumatic and grade 2 type of ACL injury	Traumatic and grade 3 type of ACL tear	Traumatic and grade 2 type of ACL tear
Onset and mechanism	Fell on the flexed knee during a kabaddi tournament	Fell on the flexed knee in the game of shotput	The patient's injury occurred when an autorickshaw overturned, causing him to fall on an extended knee.	The patient had previously fallen from a motorbike in a road traffic accident while bending his knee.

Patient information 

Patient 1

The patient is a 20-year-old male college student who was healthy three months ago. While participating in a kabaddi tournament, he experienced a traumatic event involving a fall. During this incident, his knee was flexed at approximately 90 degrees, and he reported hearing a distinct "pop" sound. Following the injury, he immediately felt severe pain and instability in his knee, which was exacerbated by walking. The patient received initial first aid at a local hospital, where he noticed significant swelling in the knee that worsened overnight. The intensity of the pain also increased. He subsequently visited a hospital in his neighborhood where he was advised to undergo magnetic resonance imaging (MRI). The MRI findings show a grade 3 ACL tear, a partial tear of the posterior cruciate ligament (PCL), and a longitudinal tear in the anterior portion of the medial meniscus.

Patient 2

A 23-year-old student suffered a severe knee injury during a shot put competition. While running, he stumbled and his knee forcefully bent, causing immediate and intense pain. The officer reported hearing a distinct pop and noticed rapid swelling around the knee joint. Imaging studies, including X-ray and MRI, carried out at a local hospital showed a complete ACL tear and a partial medial collateral ligament (MCL) tear.

Patient 3

A 24-year-old male student was traveling to his hometown in an autorickshaw when the vehicle collided with a divider and overturned. During the accident, the student fell onto the road with his knee extended. He heard a popping sound and immediately experienced significant swelling in his knee. The patient had difficulty moving his knee. He was brought to our hospital, where an MRI was recommended. The MRI later revealed that the patient had a grade 3 ACL tear.

Patient 4

A student was injured in a motorcycle accident while traveling to his village. The accident occurred because his bike's brakes were not functioning properly, and the road was slippery due to mud. During the fall, his legs were in a flexed position, which led to immediate swelling and a feeling of instability when he attempted to walk. He was transported to our hospital for further evaluation and management. An MRI was conducted, which showed a grade 3 tear of the ACL.

Clinical examination

Prior to the commencement of the examination, we obtained the patient's informed consent for the procedure and discussed the possibility of publishing their case as a report. Once the examination began, the patients again provided informed consent. They were alert, cooperative, and fully oriented to their surroundings, including time, place, and personal identity. The details of the clinical examination are presented in Table 2.

**Table 2:** Clinical examination of the patients VAS: Visual Analog Scale; LEFS: Lower Extremity Functional Scale; KOOS: Knee Injury and Osteoarthritis Outcome Score; IKDC: International Knee Documentation Committee

	Patient 1	Patient 2	Patient 3	Patient 4
Chief complaint	Painful and restricted range of motion, difficulty in walking	Pain around the knee joint, a sensation of giving away while walking. Knee joint movements are restricted.	Painful movements, affected mobility of the knee joint	Pain in and around the knee joint, a feeling of instability or the knee giving way while walking, restricted movement in the knee joint
Body type	Mesomorphic	Mesomorphic	Mesomorphic	Mesomorphic
On observation	The patient is positioned supine. The injured leg is elevated and immobilized in a long knee brace. Visible swelling is present. Two arthroscopic keyholes over the medial and lateral aspects of the patella.	The patient is lying on their back. The injured leg is elevated and secured in a long knee brace. Noticeable swelling present. Two arthroscopic incisions are visible on the medial and lateral sides of the patella. The skin around the knee joint is dry. No signs of atrophy are present.	The patient lies supine. The injured leg is elevated and secured in a long knee brace. Visible swelling indicating inflammation around the knee. Two small arthroscopic incisions on the medial and lateral sides of the patella. Keyholes suggest a recent arthroscopic procedure on the knee.	The patient is lying on their back. The injured leg is elevated and immobilized in a long knee brace. Noticeable swelling around the knee. Two arthroscopic incisions are visible on the inner and outer sides of the patella. Incisions suggest a recent arthroscopic procedure for the knee injury.
On palpation				
1. Swelling	Significant swelling detected	Mild swelling present around the patella	Visible swelling indicating inflammation around the knee	Noticeable swelling around the knee
2. Temperature	Normal warmth, no signs of increased inflammation	Normal temperature, no evidence of increased inflammation	Slightly increased warmth over the patella, consistent with inflammation	Slightly increased warmth, indicative of inflammation
3. Crepitus	No crepitus felt during palpation	Mild crepitus felt on patellar movement	No crepitus felt on patellar movement	Mild crepitus felt on patellar movement
4. Tenderness	Grade 3 tenderness is seen over the patella's medial side.	There is moderate tenderness on the medial aspect of the patella.	The medial aspect of the patella exhibits grade 3 tenderness.	There is significant tenderness, graded as level 2, on the inner side of the patella.
5. Patellar tracking	Smooth patellar movement during knee motion	Slight lateral deviation observed during knee flexion	Slight lateral deviation observed during knee flexion	Smooth patellar movement, no deviations observed during knee flexion
VAS	7.3	8.4	6.7	8.0
LEFS	20%	22.4%	23%	20%
KOOS	18%	20%	20%	22%
IKDC	30%	32%	27%	30%

Investigation

Before the surgery, the patients underwent a comprehensive set of evaluations, including a complete blood count, kidney function test, and liver function test. All results were within typical ranges, indicating that there were no systemic problems. Each patient had a pre-surgery MRI to confirm the ACL tear, providing crucial details on the injury’s extent and guiding the surgical approach. This is depicted in Table 3.

**Table 3:** Investigation results PDFS: proton density with fat suppression; MRI: magnetic resonance imaging

Investigation	Patient 1	Patient 2	Patient 3	Patient 4
MRI	PDFS hyperintense signal is noted in the anterior cruciate ligament, suggestive of a whole-thickness tear.	The increased signal seen along the anterior cruciate ligament represents strain.	A complete tear of the mid fiber of the anterior cruciate ligament is noted with the rest of the fibers appearing bulky with altered signal intensity and laxed.	Intrasubstance hyperintensities, partial fiber disruption, and minor sagging in the anterior cruciate ligament indicate a low-grade injury.

**Figure 1:**
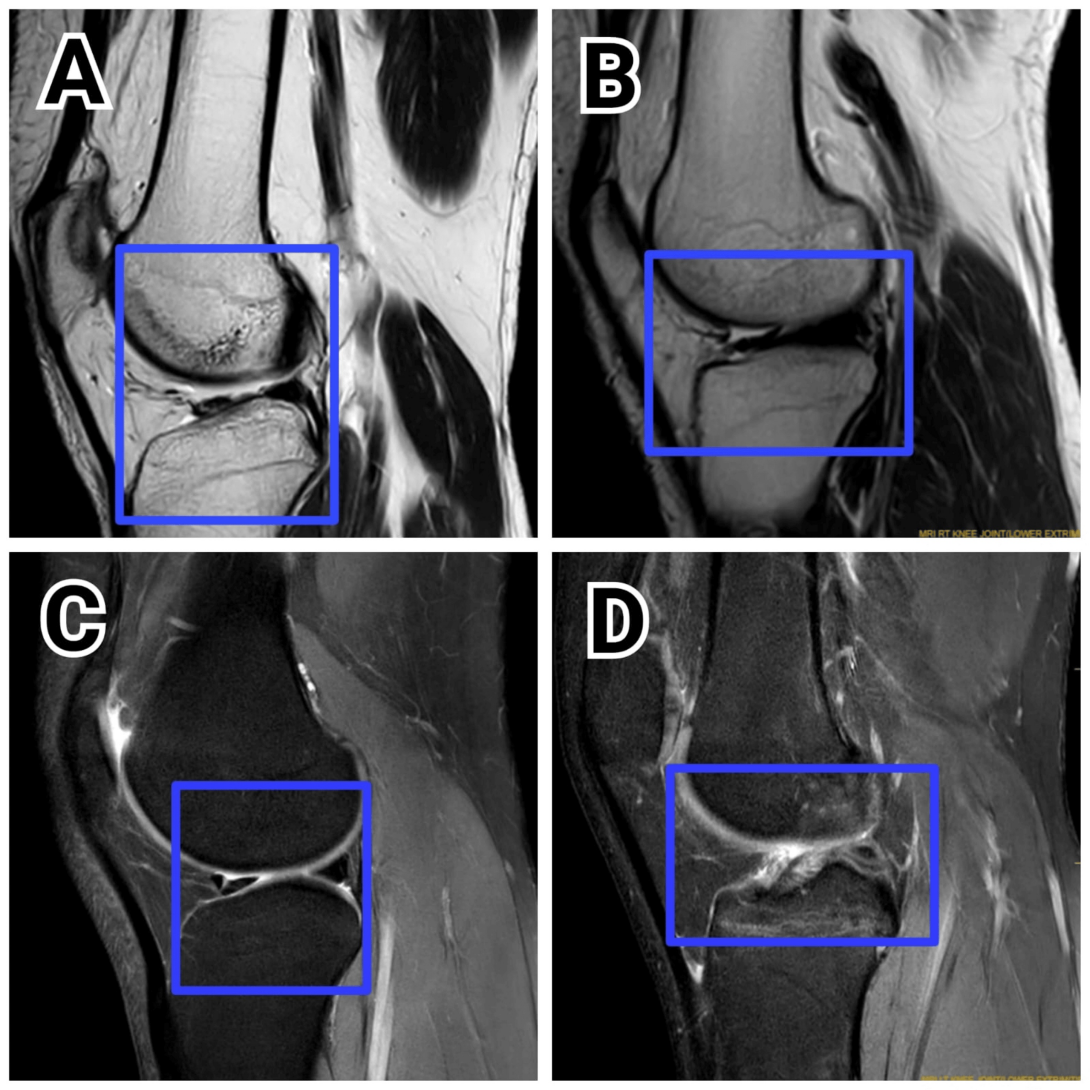
Preoperative MRI of all the four patients A: Patient 1; B: Patient 2; C: Patient 3; D: Patient 4 The highlighted region shows a tear in the anterior cruciate ligament.

Figure 1 displays the pre-surgery MRI images of all patients who underwent ACLR. These images reveal the extent of ACL injuries before surgery, aiding in the confirmation of diagnosis and surgical planning. They serve as a baseline for assessing post-surgical recovery.

**Figure 2:**
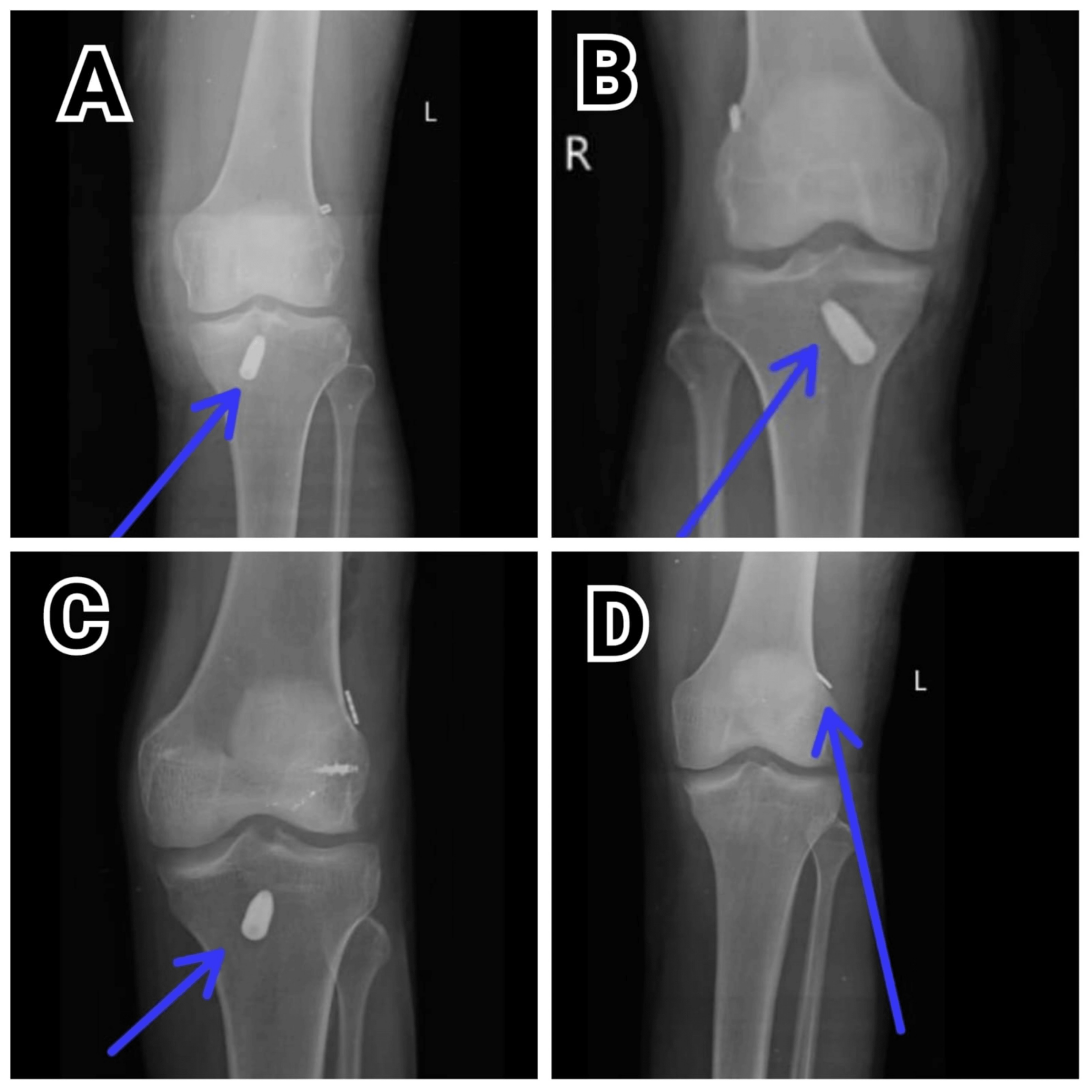
Post-operative X-rays of all the four patients A: Patient 1; B: Patient 2; C: Patient 3; D: Patient 4 The anteroposterior (AP) view of the X-ray shows the reconstruction of the anterior cruciate ligament.

Figure 2 presents post-surgery X-ray images following ACLR. These images highlight the placement of the graft and surgical hardware, as well as the alignment of the knee joint. They are essential for assessing the success of the surgery and guiding post-operative recovery. 

Diagnostic assessment

On the first day after knee surgery, patients typically experience severe pain with Visual Analog Scale (VAS) scores around 7.3, 8.4, 6.7, and 8.0, respectively, and noticeable knee swelling. Initial assessments focus on measuring the range of motion (ROM) in knee flexion and extension, often limited due to pain and swelling, and evaluating muscle strength through manual muscle testing (MMT), which usually shows reduced strength. Postoperatively, MMT is conducted in a sitting and side-lying position for the hip, prone for the knee, and standing for the ankle. Functional outcome measures include the Knee Injury and Osteoarthritis Outcome Score (KOOS) for assessing pain, symptoms, daily living activities, sports function, and quality of life; the International Knee Documentation Committee (IKDC) for evaluating basic functional mobility and balance; and the Lower Extremity Functional Scale (LEFS) for determining the patient's ability to perform everyday tasks. These evaluations establish a baseline for guiding initial rehabilitation and monitoring recovery progress. The quality of movement of all joints was painful and incomplete on day 1 of the assessment. Table 4 depicts the active ROM of the knee joint on day 1 and day 14 after surgery.

**Table 4:** Range of motion pre-rehabilitation and post-rehabilitation

Joint	Patient 1		Patient 2		Patient 3		Patient 4	
	Pre	Post	Pre	Post	Pre	Post	Pre	Post
Hip flexion	0-20°	0-70°	0-10°	0-60°	0-30°	0-80°	0-35°	0-70°
Hip extension	0-10°	0-20°	0-8°	0-15°	0-15°	0-15°	0-20°	0-20°
Hip abduction	0-30°	0-40°	0-20°	0-45°	0-20°	0-40°	0-15°	0-40°
Hip adduction	0-30°	0-40°	0-20°	0-45°	0-20°	0-40°	0-15°	0-40°
Knee flexion	0-30°	0-90°	0-25°	0-80°	0-30°	0-100°	0-35°	0-70°
Knee extension	30°-0	90°-0	25°-0	80°-0	30°-0	100°-0	30°-0	70°-0
Ankle dorsiflexion	0-15°	0-20°	0-15°	0-20°	0-20°	0-20°	0-15°	0-25°
Ankle plantar flexion	0-20°	0-25°	0-15°	0-20°	0-15°	0-20°	0-15°	0-25°

Table 5 depicts the MMT evaluation before and after rehabilitation this means the table shows muscle strength assessments using the modified Medical Research Council (MMRC) grading system. This evaluation was done both before and after the patients underwent rehabilitation.

**Table 5:** Manual muscle testing 1: Tendon becomes protruding or frail contraction experienced in muscle with no visible movement. 2-: Movement through partial range of motion. 2: Movement through complete range of motion for the muscle being tested in a gravity-eliminated plane. 2+: Holds against slight pressure in test position, moves through partial range of motion against gravity. 3-: Gradual release from the test position occurs. 3: Holds test position (with no added pressure) against gravity. 3+: Holds test position against minor pressure. 4-: Holds test position against minor to moderate pressure. 4: Holds test position against moderate pressure. 4+: Holds test position against moderate to strong pressure. 5: Holds test position against strong pressure (normal).

Joint	Patient 1		Patient 2		Patient 3		Patient 4	
Muscles of the lower limb	Pre	Post	Pre	Post	Pre	Post	Pre	Post
Hip flexors	2+	3+	2	3+	2+	4	2+	3
Hip extension	2+	3-	2	3	2	3+	2+	2+
Hip abduction	2+	3+	2	3	2	3+	2+	2+
Hip adduction	2+	3+	2	3	2	3+	2+	3-
Knee flexion	2+	3	2	3+	2+	3-	2	2+
Knee extension	2+	3	2	3+	2+	3-	2	2+
Ankle dorsiflexion	2	4+	2	4+	2	4+	2+	4
Ankle plantar flexion	2	4	2	4+	2	4+	2+	4

Physiotherapy intervention

We implemented various postoperative protocols from different guidelines over two weeks to evaluate their efficacy in patients following ACLR. By systematically applying each protocol and closely monitoring patient progress, pain levels, range of motion, quadriceps strength, weight-bearing capacity, functional status, and overall recovery, we aim to identify the most effective approach for optimizing the outcomes. This comparative analysis will provide valuable insights into which protocol best supports rehabilitation; ensuring patients receive the highest standard of care tailored to their specific needs. Table 6 denotes phase 1 rehabilitation protocols for ACLR: patient-specific guidelines and goals.

**Table 6:** Rehabilitation protocol in phase 1 POD: postoperative day; CPM: continuous passive movement

Rehabilitation procedure	Patient 1	Patient 2	Patient 3	Patient 4
Guideline	Oxford Knee Service [10]	Mass General Brigham [11]	Fowler Kennedy Sports Medicine [12]	Schlechter Protocol of Youth Sports and Ortho [13]
Time duration	POD 0-1 week	POD 0-2 weeks	POD 0-1 week	POD 0-2 weeks
Goals	This phase focuses on enhancing muscle strength and further improving movement toward full recovery.	Preserve the graft as patellar mobility is restored, discomfort and swelling are decreased, and complete extension is achieved with progressive flexion improvement. Reduce the inhibition of arthrogenic muscles, restore control over the quadriceps, and achieve complete active extension.	Control swelling and pain, protect the graft, achieve full knee extension, improve flexion, use CPM as prescribed, manage weight-bearing, minimize quadriceps inhibition, and attend physical therapy as needed.	Educate on weight-bearing status, adjust for concurrent pathologies, and focus on reducing pain and swelling, improving range of motion, and maintaining flexibility, quadriceps activation, balance, and cardiovascular fitness.

**Figure 3:**
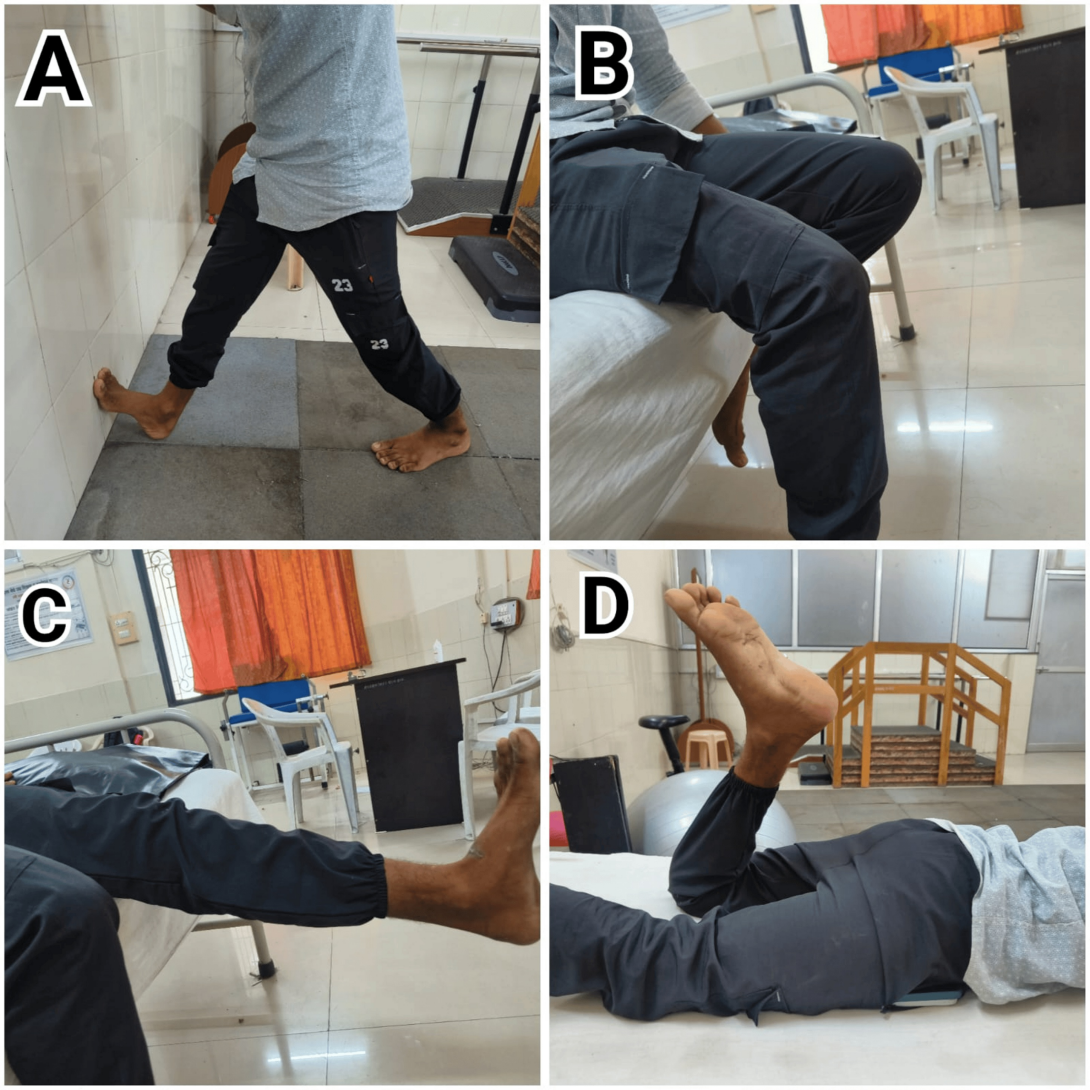
Patient 1 performing exercises from the Oxford Knee Protocol A: Wall calf stretch the patient performs a calf stretch against the wall to improve flexibility and range of motion in the lower leg. B: Seated knee flexion the patient sits on a bed or chair and performs knee flexion exercises to enhance joint mobility and flexibility. C: Straight leg raise the patient performs a straight leg raise to strengthen the quadriceps and support knee stability. D: Prone knee flexion the patient lies prone and performs knee flexion exercises to strengthen the hamstrings and improve knee function.

Table 7 denotes rehabilitation protocols in phase 1 for ACLR: criteria, interventions, and dosage. The table outlines the structured approach for ACL reconstruction rehabilitation. It specifies the criteria for progressing through stages, the therapeutic interventions employed, and the dosage, including frequency, duration, and intensity of these interventions to ensure effective recovery. Figure 3A-3D denotes patient 1 doing the exercises from the protocol given by the Oxford Knee Service.

**Table 7:** Rehabilitation protocol of phase 1 after anterior cruciate ligament reconstruction (ACLR) NMES: neuromuscular electrical stimulation; LEFS: lower extremity functional score; ROM: range of motion; HEP: home exercise program

Category	Patient 1	Patient 2	Patient 3	Patient 3
Criteria for progression	Not mentioned	Extension to 0 degrees, patellar glide, quadriceps contraction, leg elevation without stuttering.	Minimal swelling, full passive extension, partial weight-bearing with crutches, strong quadriceps contraction.	LEFS: 32-50
Intervention and dosage	Calf exercises	Swelling management	ROM/mobility	Strengthening
	Standing calf raises: 3 sets of 15, 2 times per day.	Ice: 20 min every 2 hours. Compression: Use as advised. Elevation: Leg above heart level.	Patellar mobilizations: 3-5 min, 3-4 times per day. Heel slides: 2-3 sets of 10-15 reps, 3-4 times per day. Bike pendulums: 5-10 min, 2-3 times per day.	Quadriceps/hamstrings: Co-contractions, isometrics, sit to stand, lunges, mini wall squats: 2-3 sets of 10-15 reps, 3-4 times per day.
-	-	Retrograde massage: 5-10 min, 2-3 times per day. Ankle pumps: 10-20 reps every 2 hours while awake	Leg extensions/prone hangs: 2-3 sets of 10-15 reps, 3-4 times per day. Calf/hamstring stretches: Hold for 30 sec, 2-3 times per day.	Hip/gluteal: Abduction/adduction, gluteal squeezes, hip extensions/flexions: 2-3 sets of 10-15 reps, 3-4 times per day.
Strengthening	Calf raises: 2-3 sets of 10-15 reps, 3-4 times per week.	NMES: 10 sec per 30 sec, 10 contractions per session, 2 times per week. Straight leg raise: 2-3 sets of 10-15 reps, 3-4 times per day.	Calf raises: 2-3 sets of 10-15 reps, 3-4 times/wk. Quadriceps sets: 2-3 sets of 10-15 reps, 3-4 times per day. Hip abduction: 2-3 sets of 10-15 reps, 3-4 times/wk.	Calves: Ankle pumps hourly. Standing calf raises: 2-3 sets of 10-15 reps, 3-4 times per day.
Proprioception	Not included	Single leg stance: 30-60 sec, 2-3 times per day. Wobble boards: 2-3 sets of 10-15 reps, 3-4 times per day.	Single leg stance: 30-60 sec, 2-3 times per day. Wobble boards: 2-3 sets of 10-15 reps, 3-4 times per day.	Not included
Gait training	Not included	Weight shifting: 2-3 sets of 10-15 reps, 3-4 times per day. Crutches: Gradually transition to independent gait.	Weight shifting: 2-3 sets of 10-15 reps, 3-4 times per day. Crutches: Gradually transition to independent gait.	Not included
Modalities	Not included	Ice: 15-25 min, 3-4 times per day. Interferential current therapy: As advised. Muscle stimulation: As advised.	Ice: 15-25 min, 3-4 times per day. Interferential current therapy: As advised. Muscle stimulation: As advised.	Not included
Additional interventions	Not included	Seated calf stretches: Hold for 30 sec, 3 times per leg, twice daily. Static knee bends: 3 sets, twice daily.	Static hamstring stretches: Hold for 30 sec, 3 times per leg, twice daily. Dynamic hamstring curls: 3 sets of 15 reps, once daily.	Standing knee bends: 3 sets of 10-15 reps, twice daily. Teach HEP and educate patient about their surgery.

**Figure 4:**
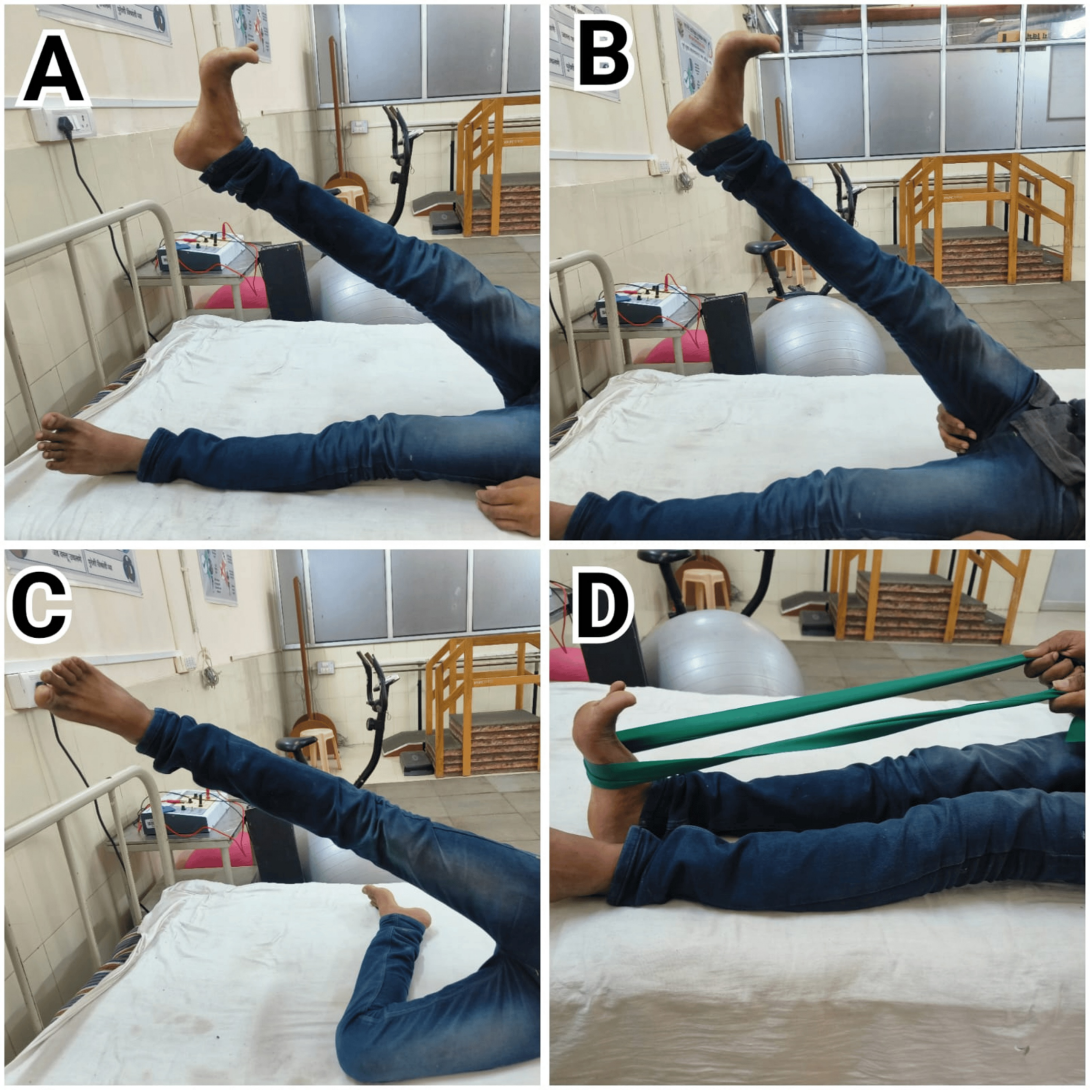
Physiotherapy exercise regimen by the Mass General Brigham given to pateint 2 A: Straight leg raise; the patient performs a straight leg raise to strengthen the quadriceps and improve knee stability. B: Active assisted knee flexion the patient engages in active assisted knee flexion exercises to increase range of motion and flexibility. C: Straight leg raise with external rotation the patient performs a straight leg raise with external rotation to target different muscle groups around the knee. D: Self calf stretching with band.

Figure 4A-4D illustrates rehabilitation exercises for ACLR as per the guidelines of the Mass General Brigham, which was given to patient 2. These figures showcase various stages and specific exercises designed to aid recovery post-surgery.

**Figure 5:**
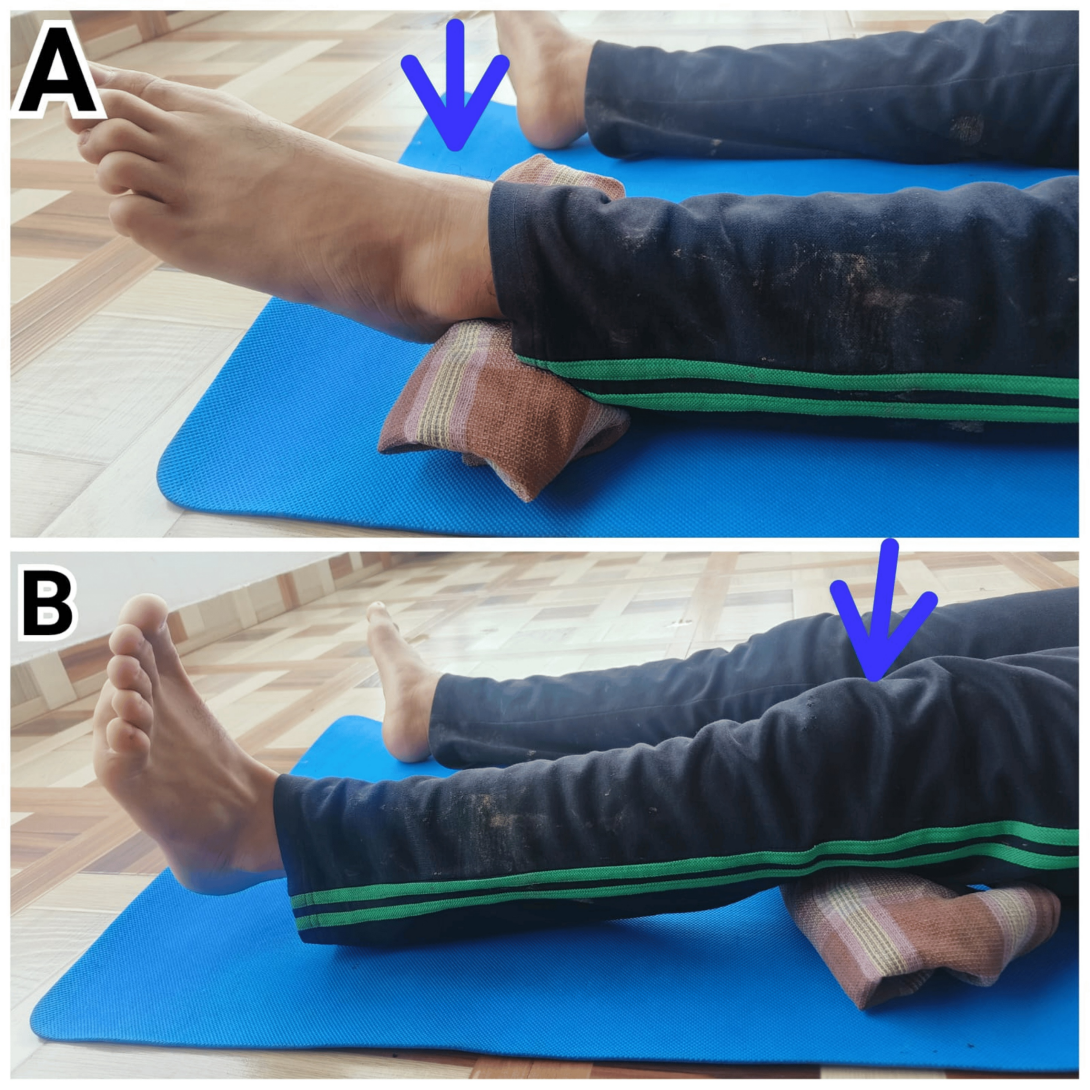
Patient 3 performing exercise from the Fowler Kennedy exercise protocol A: Quadriceps set for strengthening quadriceps muscles. B: Hamstring set for strengthening hamstring muscles.

Figure 5 demonstrates an exercise from the Fowler Kennedy Sports Medicine guidelines, which focuses on injury prevention and rehabilitation. This exercise is designed to improve strength and functionality in patients.

**Figure 6:**
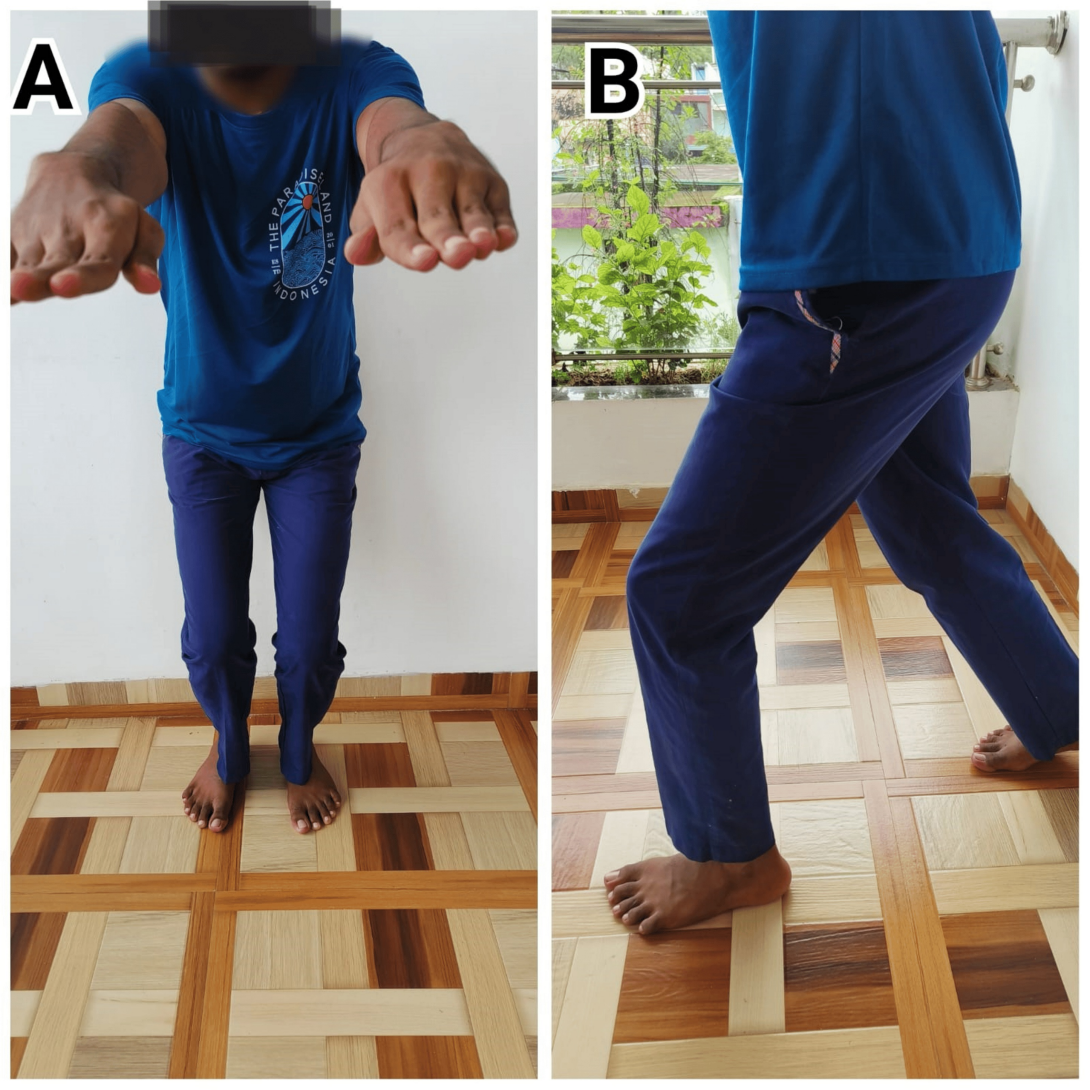
Patient 4 performing exercises from the Schlechter Protocol of Youth Sports and Ortho guidelines A: Mini squats. B: Forward lunges.

Figure 6 illustrates a specific exercise from the Schlechter Protocol of Youth Sports and Ortho guidelines, designed to enhance athletic performance and reduce injury risk. This exercise is likely targeted at improving strength, flexibility, or stability in patients.

Results of the study

The case series analysis revealed varied outcomes in ROM and MMT among patients with ACL tears. The post-rehabilitation assessments demonstrated a range of recovery outcomes among the patients involved in the study.

ROM Findings

Patient 3 exhibited the most significant improvement, achieving the highest range of motion in knee flexion (0-100°) and extension (100°-0), as well as favorable hip flexion (0-80°). Patient 4 showed the least improvement, especially in knee flexion and extension (0-70° for both), indicating a need for further intervention. Patient 1 and patient 2 displayed moderate recovery levels with varied results across different joints.

MMT Results

In MMT assessments, patient 3 had the highest muscle strength scores, particularly in hip flexion (4), while patient 4 recorded the lowest scores across several muscle groups, including knee flexion (2+) and extension (2+). Patients 1 and 2 presented moderate muscle strength with scores mostly in the 3+ range.

Outcome measures

Table 8 summarizes the post-intervention outcomes for ACL reconstruction, including functional scores and pain levels. These measures evaluate the effectiveness of the rehabilitation protocols and patient recovery progress.

**Table 8:** Outcome measure VAS: Visual Analog Scale; IKDC: International Knee Documentation Committee; KOOS: Knee Injury and Osteoarthritis Outcome Scale; LEFS: Lower Extremity Functional Scale

Outcome measure	Patient 1	Patient 2	Patient 3	Patient 4
VAS	4.8	5.0	3.8	5.7
IKDC	43.7%	48.3%	50.7%	40.2%
KOOS	26%	34%	48%	36%
LEFS	56.8%	40%	68%	38%

The VAS scores indicated varying pain levels post-rehabilitation, with patient 3 reporting the least pain (3.8) and patient 4 the highest (5.7). In the IKDC score, patient 3 outperformed the others with a score of 50.7%, while patient 4 scored the lowest at 40.2%. The KOOS percentages reflected similar trends, with patient 3 achieving the highest percentage (48%) and patient 4 the lowest (36%). The LEFS also highlighted varying functional recoveries, where patient 3 scored highest at 68%, compared to patient 4’s 38%. These findings suggest that patient 3 experienced superior functional recovery and rehabilitation outcomes, while patient 4 may benefit from more tailored rehabilitation strategies to enhance recovery. The contrasting levels of improvement among patients underscore the importance of individualized rehabilitation approaches for optimal results.

## Discussion

This case series study investigated the efficiency of numerous new rehabilitation methods and strategies post-ACLR in four patients. The outcomes suggest that while all patients showed improvements, the rate and extent of progress varied significantly, highlighting the potential influence of individualized factors and specific rehabilitation protocols [14]. Patient 3 demonstrated the most significant improvement in ROM, muscle strength, and functional measures, suggesting that the rehabilitation protocol followed by this patient may be particularly effective. This protocol, derived from the Fowler Kennedy Sports Medicine guidelines, emphasized early mobilization, aggressive strengthening exercises, and consistent physiotherapy sessions. The superior outcomes in patient 3 underline the importance of a well-structured and rigorous rehabilitation program. Conversely, patient 4, who followed the Schlechter Protocol of Youth Sports and Ortho guidelines, showed the least improvement. This protocol focused more on gradual progression and conservative management in the initial stages post-surgery. The relatively slower recovery in patient 4 suggests that while a cautious approach may prevent re-injury, it might also delay the rehabilitation process. According to Jenkins et al., rehabilitation strategies are increasingly patient-dependent, utilizing new modalities to accelerate recovery, and emphasizing the psychological component of return to sport, with positive preliminary findings [15].

Early mobilization is an important aspect of successful ACL recovery. Patients 1 and 3, who began weight-bearing and range-of-motion exercises earlier in their rehabilitation programs, showed faster improvements in knee flexion and extension. This finding aligns with existing literature that advocates for early mobilization to enhance joint function, reduce stiffness, and promote better overall recovery outcomes. Muscle-strengthening exercises played a pivotal role in the rehabilitation process. Patient 3's protocol included aggressive quadriceps and hamstring strengthening exercises, leading to significant improvements in muscle strength as indicated by MMT scores. Strengthening the muscles surrounding the knee joint is crucial for restoring stability and function post-ACLR [16]. The moderate progress observed in patients 1 and 2 also underscores the benefits of targeted muscle strengthening in ACL recovery. The variation in recovery outcomes across patients suggests that individual factors such as age, occupation, and the mechanism of injury may influence the effectiveness of rehabilitation protocols [17]. For instance, patient 2, a student, showed moderate improvement despite following a less intensive protocol. This could be attributed to the patient's baseline physical fitness and motivation to return to an active lifestyle. Similarly, the status of patients 1 and 3 may have contributed to their relatively faster recovery compared to patient 4, with potentially different physical demands and recovery priorities.

The diverse outcomes observed in this case series highlight the necessity for personalized rehabilitation strategies. While standardized protocols provide a framework for rehabilitation, tailoring these protocols to individual patient needs, preferences, and circumstances can optimize recovery. Future research should focus on developing adaptable rehabilitation plans that consider patient-specific factors and adjust intensity and progression accordingly [18]. This study is constrained by its small sample size, which limits the generalizability and robustness of the findings. To validate these results and draw more definitive conclusions, larger studies encompassing more diverse populations are necessary. In addition, the two-week follow-up period is brief and may not fully capture the long-term outcomes, including the potential for re-injury or complications. Extending the follow-up duration in future studies would provide a better understanding of the rehabilitation's long-term effectiveness.

Furthermore, the study primarily relied on subjective measures, such as VAS scores and patient-reported outcomes (KOOS, IKDC, and LEFS), which are susceptible to individual perception and reporting bias. Incorporating objective assessments, including biomechanical analysis and imaging, would enhance the evaluation of rehabilitation efficacy by providing more comprehensive and reliable data [19].

## Conclusions

This case series contributes valuable information on the variability in recovery after ACLR and highlights the importance of personalized rehabilitation approaches. By addressing individual patient factors and refining rehabilitation protocols, healthcare providers can enhance recovery outcomes and better respond to the specific requirements of patients with ACLR. Further research is needed to build on these findings and develop evidence-based guidelines for ACL rehabilitation.
